# Retraction: Effects of microRNA-708 on Epithelial-Mesenchymal Transition, Cell Proliferation and Apoptosis in Melanoma Cells by Targeting LEF1 Through the Wnt Signaling Pathway

**DOI:** 10.3389/pore.2021.1609917

**Published:** 2021-07-12

**Affiliations:** Frontiers Media SA, Lausanne, Switzerland

**Song, X., Wang, Q. and Huo, R. Effects of microRNA-708 on Epithelial-Mesenchymal Transition, Cell Proliferation and Apoptosis in Melanoma Cells by Targeting LEF1 Through the Wnt Signaling Pathway. Pathol. Oncol. Res. 25, 377–389 (2019). doi: https://doi.org/10.1007/s12253-017-0334-z**

The journal and authors retract the article cited above for the following reasons provided by the authors:

The authors have identified errors in Figures 9, 10, 11 and 12. The authors state “We apologize that we have to retract our paper: Effects of microRNA-708 on Epithelial-Mesenchymal Transition, Cell Proliferation and Apoptosis in Melanoma Cells by Targeting LEF1 Through the Wnt Signaling Pathway (doi: 10.1007/s12253-017-0334-z), Pathology and Oncology Research 25:377–389 (2019). In our recent cell function experiments, we found that some of the previous experimental results cannot be repeated. Compared with the Blank and NC groups, the melanoma cells in the miR-708 mimics group and miR-708 inhibitors group showed no significant difference in cell proliferation (MTT assay), migration (scratch test, transwell assay). While the melanoma cells in the miR-708 inhibitors + siRNA-LEF1 group were significantly inhibited, this result may indicate that miR-708 does not regulate the proliferation, EMT, and apoptosis of melanoma cells by targeting LEF1 through the Wnt signaling pathway. Since miR-708 may also promote tumor cells through other molecular mechanisms, the experimental results of cell function in the miR-708 interference group are not significantly different from those in the control group, which indicated that the logic of our experimental route was not rigorous enough and there may be many other branches.”

This retraction, as requested by the authors, was approved by the Editors-in-Chief of the Journal.

